# Attenuating lipid metabolism in atherosclerosis: The potential role of Anti-oxidative effects on low-density lipoprotein of herbal medicines

**DOI:** 10.3389/fphar.2023.1161657

**Published:** 2023-03-31

**Authors:** Huxinyue Duan, Pan Song, Ruolan Li, Hong Su, Lisha He

**Affiliations:** ^1^ School of Pharmacy, School of Basic Medicine, Chengdu University of Traditional Chinese Medicine, Chengdu, China; ^2^ Chengdu Integrated TCM and Western Medicine Hospital, Chengdu, China

**Keywords:** atherosclerosis, oxidative stress, lipid metabolism, low-density lipoprotein, herbal medicines

## Abstract

Atherosclerosis (AS) is a multifactorial chronic disease with great harm to the health of human being, which is a basic pathogenesis of many cardiovascular diseases and ultimately threatens human life. Abnormal blood lipid level is one of the most common diagnostic indicators of AS in clinic, and lipid metabolism disorder is often observed in patients with AS. Cholesterol is an important lipid in the human body, which is of great significance for maintaining normal life activities. Generally, cholesterol is transported to peripheral tissues by low-density lipoprotein (LDL), and then transported to the liver by high-density lipoprotein (HDL) *via* its cholesterol reverse transport function, and finally discharged. Under oxidative stress condition, LDL is commonly oxidized to the form ox-LDL, which is ingested by macrophages in large quantities and further forms foam cells, disrupting the normal metabolic process of cholesterol. Importantly, the foam cells are involved in forming atherosclerotic plaques, whose rupture may lead to ischemic heart disease or stroke. Furthermore, ox-LDL could also promote the development of AS by damaging vascular endothelium, promoting the migration and proliferation of smooth muscle cells, and activating platelets. Therefore, inhibiting LDL oxidation may be an effective way to improve lipid metabolism and prevent AS. In recent years, increasing studies have shown that herbal medicines have great potentiality in inhibiting LDL oxidation and reducing ox-LDL induced foam cell formation. Accordingly, this paper summarized current research on the inhibitory effects of herbal medicines against LDL oxidation and foam cell formation, and made a brief description of the role of cholesterol and LDL in lipid metabolism disorder and AS pathogenesis. Importantly, it is suggested that herbal medicines could inhibit LDL oxidation and regulate cholesterol homeostasis *via* downregulation of CD36 and SR-A, whereas upregulation of ABCA1 and ABCG1.

## 1 Introduction

Atherosclerosis (AS) is a common chronic inflammatory disease characterized by the accumulation of fatty substances under the vascular subintimal layer, and which is the basis of various cardiovascular diseases, such as myocardial infarction and cerebral infarction, causing a large number of deaths worldwide (Gupta et al., 2020). The pathogenesis of AS is complex and has not yet been fully elucidated. Accumulating studies have shown that abnormal lipid metabolism, oxidative stress, injury and dysfunction of endothelial cell, and hyperproliferation and migration of smooth muscle cell are involved in the development of AS (Witztum, 1994; Weber and Noels, 2011). Based on basic and clinical research, there are several popular theories about the pathogenesis of AS, including fatty infiltration, endothelial cell injury, platelet aggregation, and the oxidation hypothesis, etc. In addition, abnormal lipid metabolism is an independent risk factor for AS, mainly manifested as hyperlipidemia, which plays a vital role in the occurrence and development of AS (Gupta et al., 2020). The relationship between cholesterol (which is an important lipid in the human body) and AS has been aroused considerable concerning for a long time. Goldstein and Brown, two American scientists, who won the Nobel Prize in Medicine and Physiology in 1985, discovered the mechanism of LDL receptor, proving that plasma LDL is the direct main pathogenic factor of AS. Otherwise, an American scientist named Steinberg reported oxidation of LDL plays a crucial role in the development of AS (Steinberg, 1983).

**FIGURE 1:**
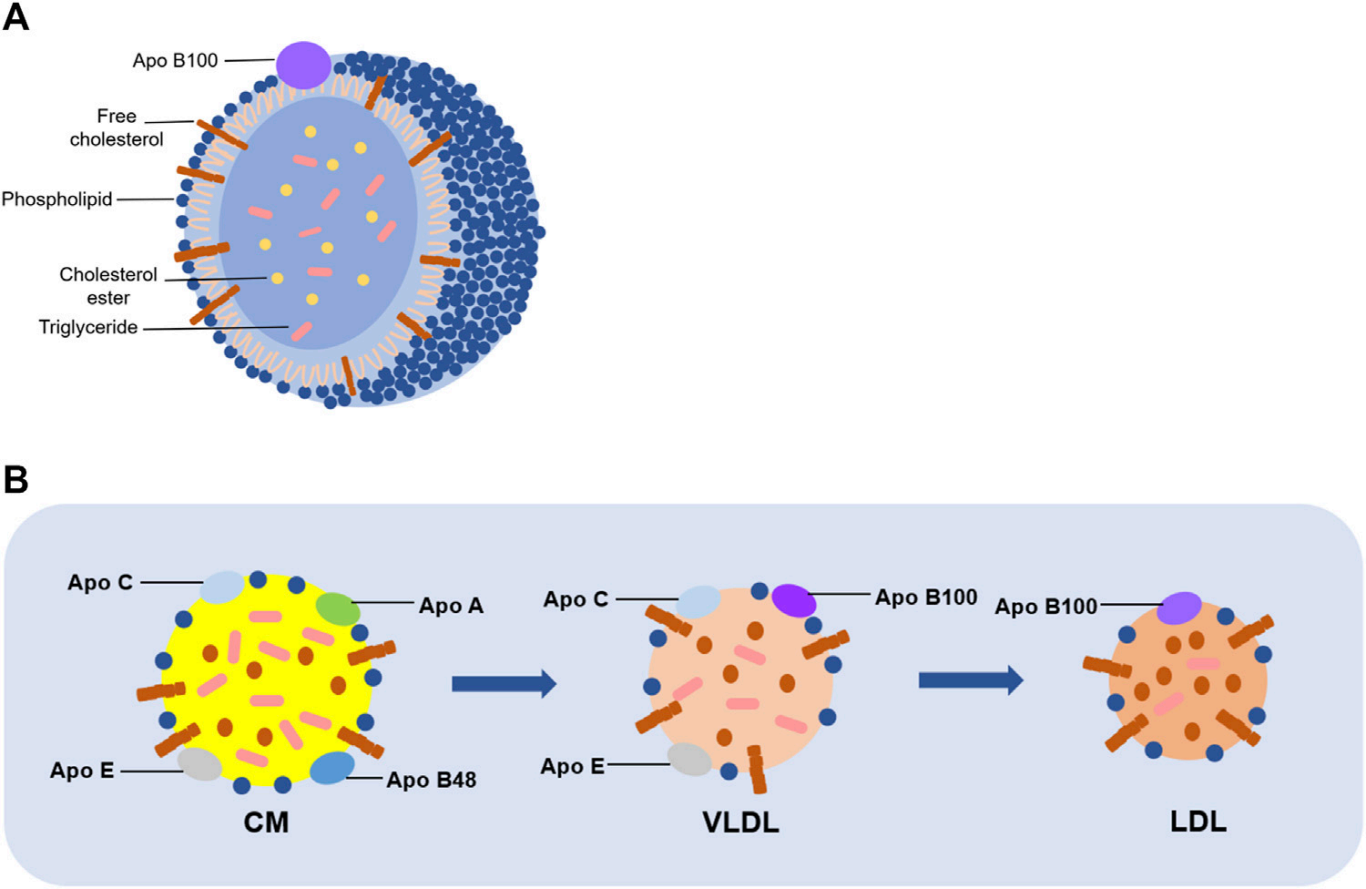
Structure and formation of LDL. **(A)** A single LDL particle is composed of phospholipids, unesterified cholesterol and Apo B in the outer layer, and cholesterol esters and triglycerides in the inner layer. **(B)** CM mainly consists of dietary fat and several kinds of apolipoprotein, including Apo B48, Apo A, Apo C, and Apo E. It is generated on the intestinal mucosa, and then hydrolyzed into chyle particles in the intestine, which will form VLDL with Apo B100. Consequently, TG in VLDL is partially hydrolyzed, as well as losing Apo C and Apo E to be converted into LDL.

LDL is the main carrier of lipids in plasma (Itabe, 1998), and appears as a spherical particle with a particle size of about 220 nm, a mass of about 3,000 kDa, and a density ranging from 1.019 to 1.063 g/mL (Zmysłowski and Szterk, 2017). And an individual LDL particle consists of a hydrophobic region (containing about 170 triglycerides and 1,500 cholesterol esters) and a hydrophilic region (containing approximately 700 phospholipid molecules, 500 cholesterol molecules, and apolipoproteins, which is mostly Apo B) (Esterbauer et al., 1992) (Figure 1A). Importantly, it has been clarified that LDL is converted from VLDL. Briefly, CM, consisted mainly of dietary fat and Apo B48, is hydrolyzed by lipoprotein lipase and then produces a chylomicron remnant, which can be taken up by the liver. Subsequently, lipids components obtained from chylomicron remnant are recombined with Apo B, which is synthesized in liver, to form VLDLs. Then, VLDLs are released into circulation. After interaction with lipases, TG in VLDLs is partially hydrolyzed, leading to decreasing in TG and loss of Apo C and Apo E in VLDLs. As a result, the density, diameter, and composition of VLDLs are changed to convert to LDL (Matsuura et al., 2006) (Figure 1B).

Under normal physiological conditions, the human body maintains redox homeostasis. However, under the stimulation of some exogenous or endogenous factors, the production of ROS increases, resulting in redox imbalance and oxidative stress (Winterbourn, 2008; D'Autréaux and Toledano, 2007). Lipids, especially phospholipids and cholesterol esters in LDL, are prone to be oxidized under the existence of ROS, and finally generate oxidized LDL (ox-LDL). Ox-LDL is closely involved into the development of AS in many ways, such as damaging vascular endothelial cells, participating in the formation of foam cells, promoting the migration and proliferation of vascular smooth muscle cells, and activating platelets (Khatana et al., 2020). It is reported that LDL without oxidation modification does not promote the occurrence of AS, suggesting that ox-LDL is the direct risk factor (Quinn et al., 1985). The formation of ox-LDL has a significant impact on the metabolism of cholesterol. Generally, cholesterol in LDL can be absorbed by peripheral cells, and then transported by HDL to the liver for metabolism and LDL can be finally excreted to keep the normal level of cholesterol (Altmann et al., 2004; Duffy and Rader, 2009; Goldstein and Brown, 2009). This process is of great significance to ensure normal physiological activities of human being. However, once ox-LDL is formed in large quantities, excessive ox-LDL will be ingested by macrophages, resulting in ox-LDL accumulated in macrophages and could not be excreted. As a result, a large number of foam cells are formed and accumulated in the blood vessels, accelerating the development of AS (Kume et al., 2000). Therefore, inhibiting LDL oxidation is a feasible way to inhibit the formation of foam cells and improve cholesterol metabolism.

Herbal medicines have been used for treatment and prevention of various diseases for thousands of years worldwide. Accumulating evidence has shown that many herbal medicines, as complementary and alternative therapies, have beneficial effects on AS, such as red sage root (*Radix Salvia miltiorrhiza*), garlic, celery, and ginkgo (*Ginkgo biloba* L.), which exhibit the hypolipidemic effect (Hao et al., 2017). In addition, some active compounds isolated from herbal medicines, such as quercetin, resveratrol, lycopene and epigalactin-3-gallate (Zhang et al., 2021), have also been proved to have inhibitory effects on LDL oxidation. However, there is lack of systematic review on the anti-atherosclerotic effects of herbal medicines *via* regulating lipid metabolism and inhibiting LDL oxidation. Therefore, this review summarized herbal medicines used to protect AS by inhibiting LDL oxidation and lipid metabolism to provide a reference for the research of AS prevention and treatment.

## 2 Lipid metabolism disorder and lipoprotein in atherosclerosis

It is well known that normal lipid metabolism is important for humans’ health, and when lipid metabolism is abnormal, the body will undergo pathological changes. At present, lipid metabolism disorders have been regarded as one of the independent risk factors for cardiovascular diseases and have attracted great attention (Kimura et al., 2010). What is lipid metabolism disorder? It means processes of synthesis, breakdown, digestion, absorption, and transport of lipids in the body do not proceed normally. As a result, abnormal levels of lipids and their metabolites in the blood or organs usually cause hyperlipidemia, which is one of the pathogenic factors of AS (Hurtubise et al., 2016). Lipids in blood include cholesterol, triglycerides, and phospholipids, which are hydrophobic substances that must bind to apolipoproteins to form lipoproteins to be transported and metabolized. Lipoproteins include chylomicrons (CM), very low-density lipoprotein (VLDL), intermediate-density lipoprotein (IDL), low-density lipoprotein (LDL), and high-density lipoprotein (HDL) (Segrest et al., 2001; Afonso and Spickett, 2019). Among them, abnormal HDL and LDL levels are characteristic of dyslipidemia, which is clinically manifested by elevated low-density lipoprotein cholesterol levels and decreased high-density lipoprotein cholesterol levels (Gupta et al., 2020).

**FIGURE 2:**
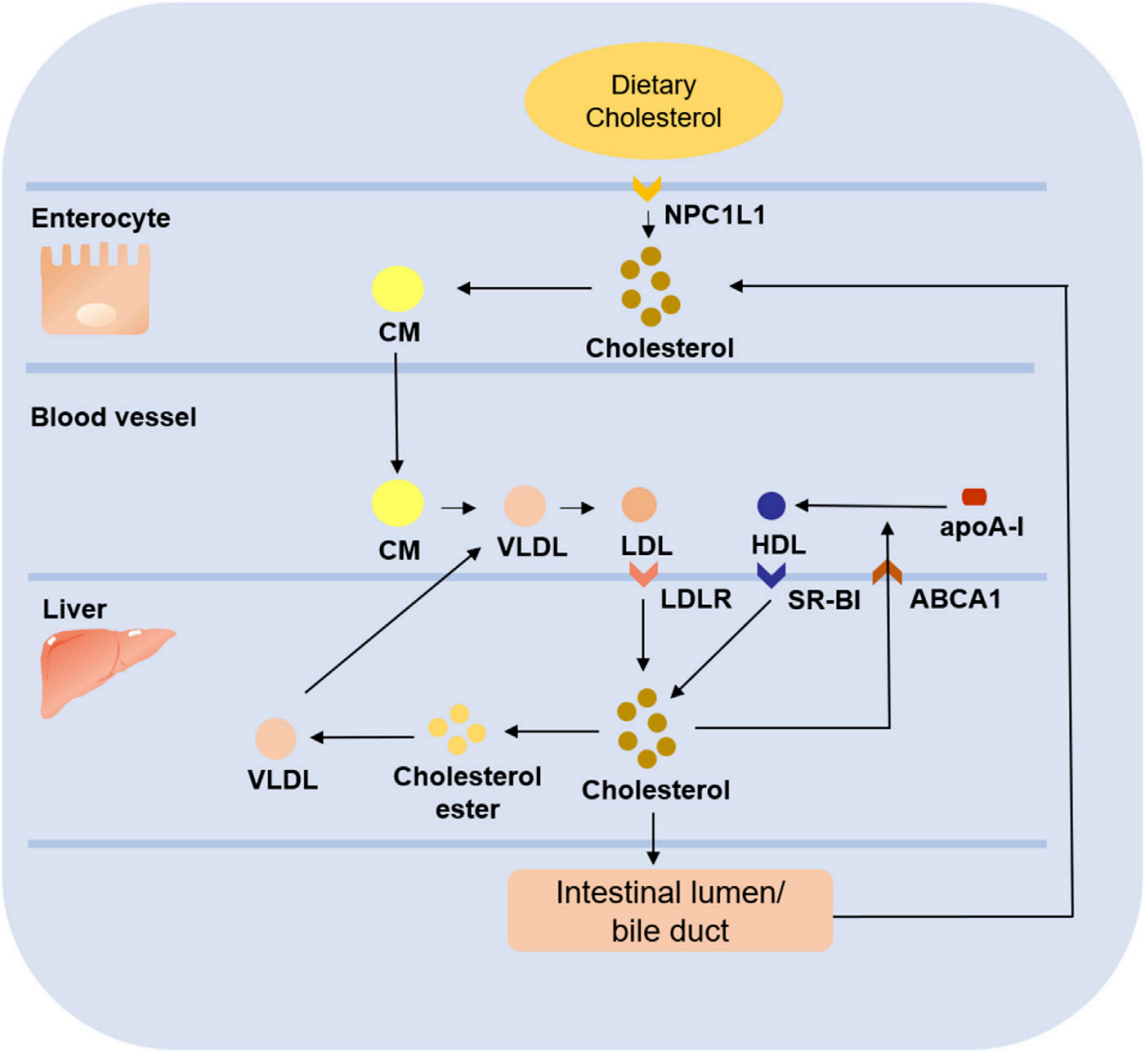
Role of LDL and HDL in cholesterol metabolism. Dietary cholesterol ingested from food enters enterocyte under the mediation of NPC1L1 and forms CM. After being released into blood, CM is gradually converted into VLDL, and then further converted into LDL, which is absorbed by liver under the mediation of LDLR. Some of the cholesterol in the liver is esterified to form cholesterol ester to form VLDL which will be released into blood, while the other part enters intestinal lumen/bile duct to be reabsorbed into the circulation or excreted from the body. In addition, the free cholesterol transferred to plasma under the mediation of ABCA1 combines with apoA-I to form nascent HDL, which is absorbed by liver through SR-BI to complete the reverse cholesterol transport.

HDL is often considered atheroprotective because of its reverse cholesterol transport (RCT) role. It lowers plasma cholesterol levels by transporting peripheral cholesterol to the liver, where cholesterol is excreted into the bile as a prototype or converted to bile acids, which are partially reabsorbed in the intestine and the remainder excreted in the feces (Duffy and Rader, 2009). In contrast, LDL transports cholesterol to peripheral tissues, and the level of cholesterol it carries is positively associated with the development of cardiovascular disease. Briefly, cholesterol ingested from food is absorbed in the intestine mediated by the Niemann-Pick type C1-like 1 (NPC1L1) protein and then released as CMs, and the cholesterol is subsequently taken up by the liver. Upon arrival in the liver, cholesterol is released into the blood as VLDLs, then converted to LDLs, which are taken up by peripheral cells mediate (Altmann et al., 2004; Goldstein and Brown, 2009) (Figure 2). Due to its small particle size, cholesterol-rich LDL can pass through vascular endothelial cells and bind to subendothelial glycoproteins to deposit on the vessel wall. The higher the concentration of LDL in the blood, the faster it deposits (Weber and Noels, 2011). Meanwhile, under the effect of oxidative stress, LDL bound to glycoproteins is easily modified to ox-LDL, which is an important step in the development of AS (Braamskamp et al., 2015). In conclusion, both HDL and LDL play an important role in lipid metabolism. The former contributes to reverse transport of cholesterol to reduce the cholesterol level in plasma. At the same time, the latter mediates transport of cholesterol to peripheral tissues, and it is easy to pass through the damaged vascular endothelium to promote the occurrence and development of AS.

## 3 Oxidative stress and oxidation of LDL

### 3.1 Oxidative stress and reactive oxygen species (ROS)

Under the stimulation of external chemical or physical factors or the effects of endogenous enzymes, the imbalance between oxidation and reduction may occur, resulting in increasing production of ROS and damage to proteins, nucleic acids, and lipids (Winterbourn, 2008; D'Autréaux and Toledano, 2007). This state in which the balance between antioxidant capacity and active species is disturbed is known as oxidative stress. ROS are derivatives of oxygen molecules produced during metabolism and are continuously produced during normal physiological processes. There are two types of ROS, called free radical ROS and non-free radical ROS(Dröge, 2002). Generally, free radical ROS are unstable in nature and include superoxide (O^2-^) and hydroxyl radical (OH), which can be produced under mediation of various enzyme systems. In contrast, non-free radical ROS have more stable properties and include hydrogen peroxide (H_2_O_2_), hypochlorous acid (HOCl), and peroxynitrite (ONOO-) (Martin-Ventura et al., 2007; Duan et al., 2021). Normal levels of ROS have an important physiological function in transmitting signaling, while the production of excess ROS causes harm to organisms (Li et al., 2014).

### 3.2 Oxidative modification of LDL

It has been reported that oxidative stress is a prerequisite for pathogenesis of AS (Yang et al., 2017). Under pathological state of oxidative stress, the vascular endothelium is damaged, allowing LDL to cross it and adhere to the intima (Kruth et al., 2002). LDL attached to the intima of blood vessels is susceptible to oxidative modification by ROS to produce ox-LDL (Frei et al., 1988; Stocker and Keaney, 2004). And there has been increasing evidence suggesting that ox-LDL, but not LDL, has a role in promoting the development of AS.

**FIGURE 3:**
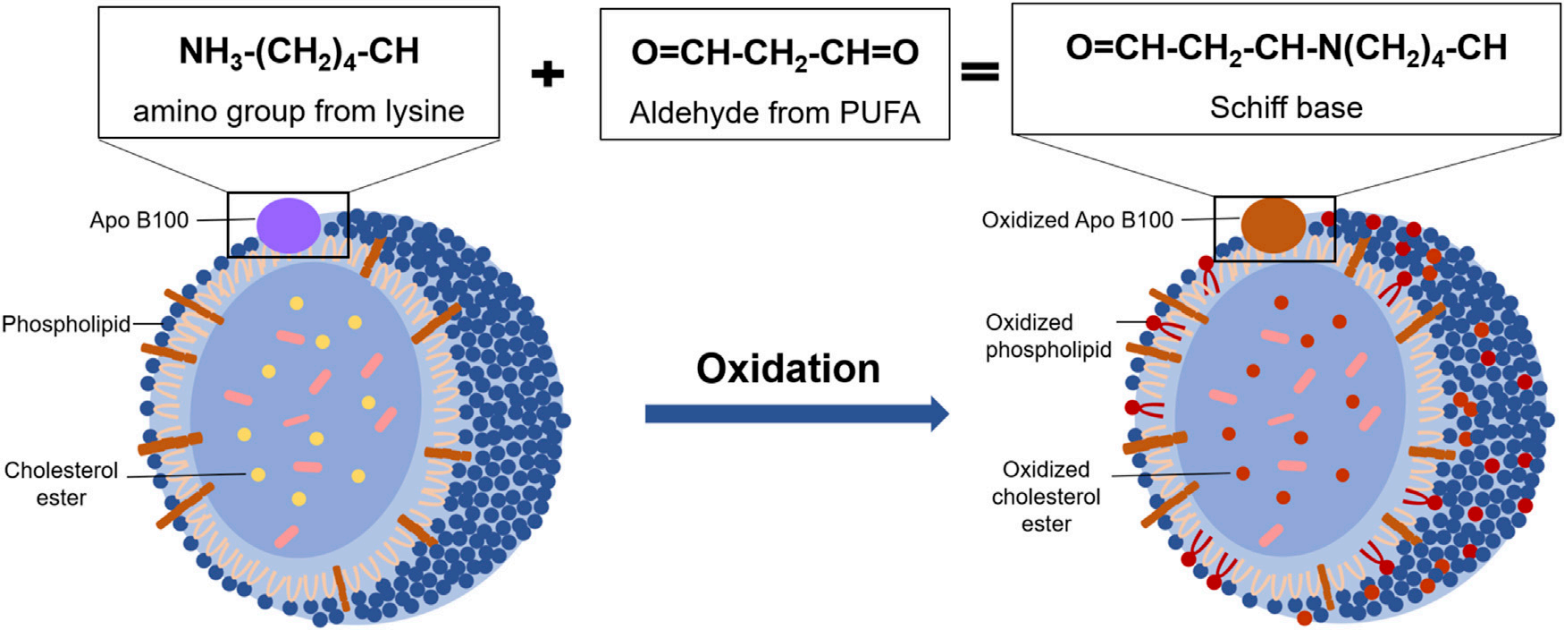
The oxidative modification of LDL. Under oxidative stress condition, phospholipids and Apo B100 in the outer layer of LDL are oxidized, and the lysine residues of Apo B100 reacted with reactive aldehydes from oxidative phospholipids to form Schiff base. Then, the cholesterol ester in the inner layer of LDL is gradually oxidized with the oxidation rate is as high as 90%.

Due to the presence of polyunsaturated fatty acid moieties, phospholipids in the outer layer of LDL are oxidized first in oxidative modification process under the presence of ROS. Then cholesterol esters in the core are oxidized gradually (Steinbrecher et al., 1990). Among them, the oxidation rate of esterified fatty acids in cholesterol esters is as high as 90% (Brown et al., 2000). In the process of LDL oxidation, a variety of bioactive molecules are formed, such as oxidized sterols, oxidized fatty acids, and some small molecules (Witztum, 1994). As a result, a large number of bioactive molecules are produced, triggering a series of subsequent reactions that cause further damage to the body. For example, cholesteryl hydroperoxy-octadecadienoate (Chol-HPODE), one of the major oxidation products of LDL, inactivates platelet-derived growth factor (Kritharides et al., 1993). In addition, Apo B100 on the surface of LDL is also highly susceptible to ROS modification and degradation to produce peptides ranging from 14 kDa to 500 kDa (Fong et al., 1987) (Figure 3). Ox-LDL adhering to the vascular endothelium recruits monocytes, which subsequently differentiate into macrophages. The CD36 receptors on the surface of macrophages recognize ox-LDL and allow it to enter macrophages. Then the retention of large amounts of ox-LDL makes macrophages form foam cells and accelerates the development of AS (Hofmann et al., 2017).

## 4 Ox-LDL in the formation of foam cells and other atherogenic ways

### 4.1 Formation of foam cells

Macrophages play an important role in cholesterol homeostasis during lipid metabolism. Under the induction of inflammatory factors and chemokines, monocytes migrate to subendothelial space through the damaged endothelium. Then, the expression of scavenger receptors and toll-like receptors on the surface of these monocytes is upregulated, marking transformation of monocytes into macrophages. There are many scavenger receptors on the surface of macrophages that mediate the uptake of lipids, such as scavenger receptor B2 (CD36), scavenger receptor class A (SR-A), and lectin-like ox-LDL receptor-1 (LOX-1). Among them, LOX-1 in macrophages is upregulated under stimulation of a variety of atherogenic stimuli, such as ox-LDL, pro-inflammatory cytokines, and advanced glycation end products (AGEs) (Kume et al., 2000; Pirillo et al., 2013). In addition, macrophages express a variety of receptors that mediate reverse cholesterol transport, including ATP-binding cassette transporter A1 (ABCA1), ATP-binding cassette protein G1 (ABCG1), and scavenger receptor class B type 1 (SR-B1) (Chistiakov et al., 2017). It means that there are different receptors that mediate lipid uptake and reverse transport of cholesterol on the surface of macrophages, and the former would be upregulated by atherogenic stimuli.

**FIGURE 4:**
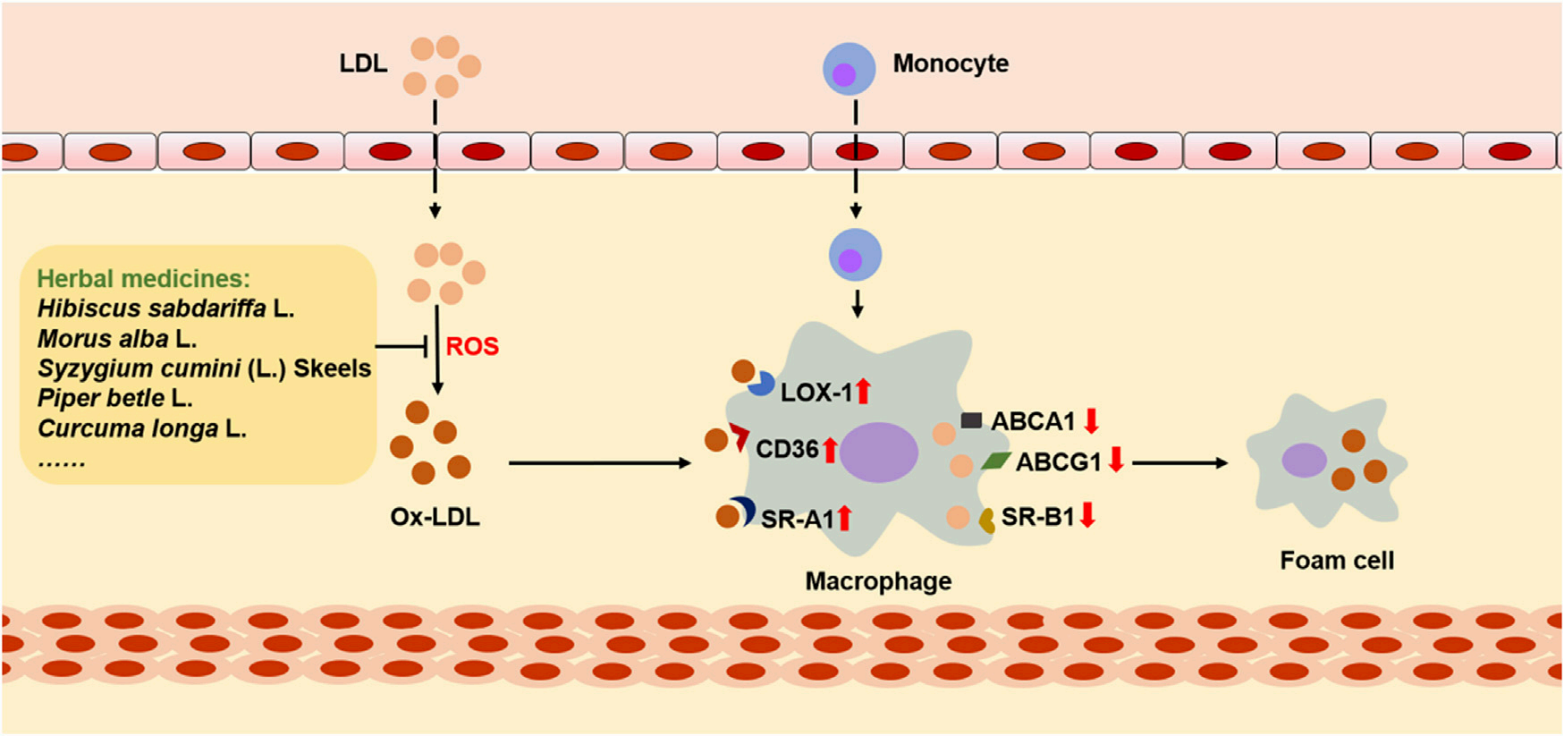
The role of ox-LDL in cholesterol metabolism and foam cell formation. LDL passes through the damaged endothelium, where it is oxidized by ROS and then converts to ox-LDL, interacting with macrophage-derived monocytes. During this process, the expression of CD36, SR-A1, and LOX-1 on the surface of macrophages is upregulated, while the expression of receptors that mediate cholesterol reverse transport is downregulated, including ABCA1, ABCG1, and SR-B1. As a result, cholesterol in macrophages accumulates and eventually foam cells are formed. However, many herbal medicines can reduce the accumulation of cholesterol and inhibit the formation of foam cells by inhibiting the oxidative modification of LDL.

In the early stage of AS, foam cells play an important role in reducing lipid accumulation, clearing apoptotic cells, and attenuating inflammation. At this stage, phagocytosis and ejection of lipids by macrophages are balanced. In brief, lipids in macrophages first react with lysosomal acid lipase (LAL) to digest cholesterol esters and produce free cholesterol, which will then be converted into cholesterol ester after treatment with acetyl-CoA acetyltransferase (ACAT1). Consequently, on the endoplasmic reticulum, these cholesterol esters will react with neutral cholesterol ester hydrolase (NCHH) to produce free cholesterol, which can be discharged by cholesterol transporters (Chistiakov et al., 2017). However, this balance will be destroyed under the influence of multiple AS risk factors. It is observed that the expression of LOX-1 is increased, and the expression of ABCA1 and ABCG1 is decreased on the surface of macrophages after the development of AS. In addition, the expression of ACAT1 is increased, while the expression of NCEH is reduced. As a result, the uptake of ox-LDL is increased. At the same time, ejection of lipids is decreased, leading to excessive accumulation of cholesterol in macrophages, which gradually makes formation of foam cells, and then accumulates into lipid stripes (Figure 4). More importantly, continuous uptake of ox-LDL by macrophages aggravates inflammation, as well as drives matrix metalloproteinase (MMP) to degrade the fibrous cap on the outer layer of the lipid cores, making AS plaque unstable or broken. Interestingly, unmodified LDL does not cause excessive accumulation of cholesterol esters by macrophages. In addition, although macrophages produce most foam cells, a few foam cells are produced by endothelial cells and vascular smooth muscle cells (Xu et al., 2021).

### 4.2 Other atherogenic ways

#### 4.2.1 Endothelial dysfunction

As a barrier between blood and the underlying layer of the vascular wall, vascular endothelium plays an important role in maintaining vascular homeostasis. Because of being exposed to circulating blood, vascular endothelium is easily susceptible to various atherogenic stimuli. Normal blood vessels regulate vascular tension to maintain stability by secreting a set of vasoactive molecules, including nitric oxide (NO), prostacyclin (PGI2), endothelium-dependent hyperpolarization factor (EDRF), and endothelin-1 (ET-1) (Xu et al., 2021). However, ox-LDL is taken up by endothelial cells under mediation of the lectin-like low-density lipoprotein receptor-1 (LOX-1), and then activates adhesion molecules in the surface of endothelial cells. As a result, monocytes migrate into the sub-endothelial layers, and then differentiate into macrophages, engulfing large amounts of ox-LDL to generate foam cells (Wang et al., 2019). What’s more, ox-LDL reduces the production of eNOS-derived NO, which is a vasodilator with protective effects on vascular endothelium (Förstermann and Sessa, 2012; Fujimura et al., 2012; Das et al., 2021). In addition, ox-LDL damages vascular endothelium in several ways, including increasing production of ROS, causing inflammation, and causing endoplasmic reticulum (ER) stress (Jiang et al., 2022). Under persistent injury, endothelial cells may experience cell death through apoptosis, necrosis, and ferroptosis, which impairs the integrity of endothelial cells, and thus aggravating endothelial dysfunction.

#### 4.2.2 Migration and proliferation of vascular smooth muscle cells

In the occurrence and development of atherosclerotic lesions, the proliferation, migration, and death of vascular smooth muscle cells (VSMCs) are involved to the formation, growth, rupture of plaque, and to vascular remodeling and occurrence of hemangioma (Alexander and Owens, 2012). Under stimulation of ox-LDL, VSMCs migrate into the subendothelial space, and then change from the contractile to the synthetic phenotype to proliferate excessively (Yang et al., 2018). It has been reported that ox-LDL-stimulation promotes VSMC proliferation by increasing the release of growth factors, including the insulin-like growth factor (IGF-1), the platelet-derived growth factor (PDGF), and epidermal growth factor (EGF), while inhibiting the expression of miR-141 (Eto et al., 2006; Sun and Chen, 2011; Goyal et al., 2012). Then, VSMCs proliferate excessively, secrete extracellular matrix proteins and synthesize collagen to form the fibrous cap for the atherosclerotic plaque. Moreover, the stability of atherosclerotic plaque is determined by the thickness of the fibrous cap. However, ox-LDL-stimulated apoptosis of VSMCs and local inflammatory reaction lead to activation of matrix metalloproteinases (MMPS), which leads to the thinning of the fibrous cap, and eventually leading to rupture of atherosclerotic plaque, which causes thrombosis in the blood vessel (Bennett et al., 2016).

#### 4.2.3 Activation of platelet

Platelets play an important role in the development of AS, with the involvement of ox-LDL. Under the stimulation of ox-LDL, platelets are rapidly activated *via* Src kinases and Rho kinase-signaling pathways (Wraith et al., 2013). In addition, the expression of P-selectin and activity of integrin α_IIb_β_3_ are increased through interaction between ox-LDL and CD36 in resting platelets, and then the expression of LOX-1 is increased (Podrez et al., 2007). As a result, LOX-1 and CD36 promote platelet adhesion to endothelial cells and increase the composition of AS plaques (Khatana et al., 2020). Additionally, chemokines released by activated platelets accelerate the development of AS by aggravating endothelial dysfunction and promoting foam cell production (Curtiss et al., 1987; Siegel-Axel et al., 2008).

## 5 Herbal medicines that inhibited oxidation of LDL and formation of foam cells

Mixtures and monomers extracted from herbal medicines are often studied. Here, aqueous extract, methanol extract, whole extract, and some monomers will be discussed (Table 1).

**TABLE 1:** Inhibition LDL oxidation and foam cell formation effects of herbal medicines in Atherosclerosis.

Botanical drugs	Part of plant used	Main components	Animal/cells	Effects	Related targets	Refs
Aqueous extract						
*Morus alba* L. [Moraceae; *Mori Folium*]	Leaf	Gallocatechin, Gallocatechin gallate, Naringenin	ox-LDL-induced J774A.1 cells	Inhibiting LDL oxidation; Inhibiting foam cell formation	PPARγ↓, CD36↓, SR-A↓, SOD-1↑, GPx↑	Yang et al. (2011)
*Syzygium cumini* (L.) Skeels [Myrtaceae; *Fructus Syzygii Cumini*]	Seed	Quercetin, Rutin, Kaempferol	ox-LDL-induced RAW264.7 cells	Inhibiting LDL oxidation; Inhibiting foam cell formation	N/A	Jadeja et al. (2012)
			Atherogenic diet-induced *Charles foster* rats	Reducing TBARS and CD contents in LDL	VCAM-1↓, P-selectin↓	
*Piper betle* L. [Piperaceae]	Leaf	3-ρ-coumaroylquinic acid, 4-ρ-coumaroylquinic acid	ox-LDL-induced J774A.1 cells	Inhibiting LDL oxidation; Inhibiting foam cell formation; Activating the reverse cholesterol transport	ABCA1↑, LXR↑	Ma et al. (2013)
**Methanol extract**						
*Hibiscus sabdariffa* L. [Malvaceae]	Flower	Cyanidin, Delphinidin	ox-LDL-induced RAW264.7 cells	Inhibiting LDL oxidation; Reducing macrophages apoptosis	Caspase-3↓	Chang et al. (2006)
	Leaf	Catechin, ECG, Ellagic acid	ox-LDL-induced J774A.1 cells	Inhibiting LDL oxidation; Inhibiting foam cell formation	LXRα↑, ABCA1↑, CD36↓, PPAR-c↓	Chen et al. (2013)
*Scoparia dulcis* L. [Plantaginaceae; *Scopariae Herba*]	Leaf	Myricetin, Rutin	ox-LDL-induced RAW264.7 cells	Inhibiting LDL oxidation; Inhibiting foam cell formation; Decreasing inflammation	N/A	Nambiar et al. (2014)
*Phyllanthus emblica* L. [Phyllanthaceae; *Phyllanthi Fructus*]	Fruit	Kaempferol, Rutin, Coumaric acid	ox-LDL-induced RAW264.7 cells	Inhibiting LDL oxidation; Inhibiting foam cell formation	N/A	Nambiar and Shetty (2015)
**Whole extract (aqueous and ethanol extract)**						
*Morus alba* L. [Moraceae; *Mori Fructus*]	Fruit	Cyanidine-3-glucoside, Cyanidine-3-rutinoside, Pelargonidin-3-glucoside, Pelargonidine-3-rutinoside	ox-LDL-induced J774A.1 cells	Inhibiting LDL oxidation; Reducing macrophages apoptosis; Inhibiting foam cell formation	N/A	Liu et al. (2008)
*Trachyspermum ammi* (L.) Sprague [Apiaceae]	Seed	2-(4-Methyl-1H-1,2,3-triazol-1-yl) ethan-1-amine, 1,3-Dioxolan-2-one, 4,5-bis (methylene)-, N-methylene-n-ctadecylamine	ox-LDL-induced RAW264.7 cells	Inhibiting LDL oxidation; Inhibiting foam cell formation	N/A	Priyaa and More (2022)
**Others**						
*Pinus morrisonicola* Hayata [Pinaceae]	Needle	1-Docosene, Neophytadiene, Methyl abietate	ox-LDL-induced RAW264.7 cells	Inhibiting lipid peroxidation; Inhibiting foam cell formation	N/A	Cheng et al. (2015)
*Citrus × aurantium f. deliciosa* (Ten.) M.Hiroe [Rutaceae]	Peel	Limonene	ox-LDL-induced RAW264.7 cells	Inhibiting LDL oxidation; Inhibiting foam cell formation; Decreasing cholesterol synthesis	CD36↓	Castro et al. (2020)
**Monomers**						
*Curcuma longa* L. [Zingiberaceae; *Curcumae Longae Rhizoma*]	N/A	Curcumin	ox-LDL-induced RAW264.7 cells	Inhibiting LDL oxidation; Inhibiting foam cell formation	CD36↓, MDA↑	Kou et al. (2013), Min et al. (2013)
*Vitex rotundifolia* L.f. [Lamiaceae]	N/A	(3R)-5-Hydroxymellein	ox-LDL-induced RAW264.7 cells	Inhibiting LDL oxidation; Inhibiting foam cell formation	N/A	Kim et al. (2020)
N/A	N/A	Maslinic acid	ox-LDL-induced THP-1 monocytes	Inhibiting LDL oxidation; Inhibiting foam cell formation; Reducing monocytes adhesion; Enhancing cholesterol efflux	ABCA1↑, ABCG1↑, VCAM-1↓, MCP-1↓, CD36↓, SR-A↓	Phang et al. (2020)
*Dendrobium venustum* Teijsm. & Binn. [Orchidaceae]	N/A	Lusianthridin	ox-LDL-induced RAW264.7 cells	Inhibiting LDL oxidation; Inhibiting foam cell formation	N/A	Thant et al. (2021)

### 5.1 Aqueous extract

#### 5.1.1 *Morus alba* L

Mulberry, the fruit of *Morus alba* L., is rich in anthocyanins, which have been reported to possess many potentially therapeutic benefits, such as anti-oxidation, anti-inflammation, and anti-tumor (Dai et al., 2007). In 2008, Liu et al. (Liu et al., 2008) investigated the effect of mulberry anthocyanin-rich extracts (MACs) on Cu^2+^-induced LDL and ox-LDL-induced macrophages. The results showed that MACs (0.1 mg/mL) could suppress the REM, Apo B fragmentation, and TBARS formation, which meant MACs had the ability to inhibit the oxidation of LDL. Then, the picture of Oil Red O staining suggested MACs reduced macrophage-derived foam cell formation. All these results suggested that MACs had an anti-oxidative effect on preventing or treating AS. Moreover, cyanidine-3-glucoside, cyanidine-3-rutinoside, pelargonidin-3-glucoside, and pelargonidine-3-rutinoside were identified from mulberry anthocyanin-rich extracts by HPLC/ESI/MS/MS.

#### 5.1.2 *Syzygium cumini* (L.) skeels

*Syzygium cumini* (L.) Skeels is a plant of the Myrtaceae family, and the flavonoid-rich fraction of its seed has been reported to have many pharmacological effects, such as lowering blood lipids, anti-diabetes, and preventing cardiac and hepatic oxidative stress. Researchers (Sharma et al., 2008; Jadeja et al., 2012) studied the effect of flavonoid-rich *S. cumini* (L.) Skeels seed extract (SSE) on Cu2^+^-induced LDL, ox-LDL-induced RAW264.7 cells and atherogenic diet (ATH)-induced Charles foster rats, and found that the main components of SSE are quercetin, rutin, and kaempferol. SSE could attenuate the oxidation of LDL *in vitro*, reduce foam cells formation, and reducing TBARS and CD contents in LDL isolated from rats. In addition, SSE ameliorated histoarchitectural changes of the thoracic aorta in ATH-induced rats and upregulated the expression of adhesion molecules *viz.* VCAM-1 and P-selectin. These results indicated that SSE might prevent AS through its anti-oxidative effects.

#### 5.1.3 *Piper betle* L

*Piper betel* L., a plant of the Piperaceae family, has been shown to have anti-oxidative activity with low toxicity *in vitro* and *in vivo* (Choudhary and Kale, 2002), but its effects on LDL oxidation and foam cell formation are unclear. Ma et al. (Ma et al., 2013) studied the influence of the extract of *Piper bete*l L. leaves (PBLs) on Cu2^+^-induced LDL and ox-LDL-induced J774A.1 cells, and explored underlying molecular mechanisms. Their results showed that the main components of PBLs are 3-ρ-coumaroylquinic acid and 4-ρ-coumaroylquinic acid, and PBLs (0.1, 0.5, and 1.0 mg/mL) inhibited the oxidation of LDL, and further reduced the uptake of ox-LDL by macrophages and, in turn, prevented the formation of foam cells. In addition, PBLs reduced the lipid accumulation in macrophages *via* increasing levels of the class A and class B scavenger receptors, ABCA1, and Liver X receptor (LXR) in ox-LDL-induced J774A.1 cells, suggesting that PBLs activated the reverse cholesterol transport to prevent both lipid accumulation and foam cell formation.

### 5.2 Methanol extract

#### 5.2.1 *Hibiscus sabdariffa* L

Due to a variety of pharmacological effects, *Hibiscus sabdariffa* L. is considered to be beneficial for health, and it possesses antioxidant components, including vitamin E, β-carotene and anthocyanins (Tsuda et al., 1996; Tsuda et al., 2000; Ramirez-Tortosa et al., 2001). In 2006, Chang et al. (Chang et al., 2006) studied the antioxidant effect of *H. sabdariffa* L. (HS), extracted from the dried flower *H. sabdariffa* L., on LDL *in vitro*. In their study, relative electrophoretic mobility (REM), fragmentation of Apo B, and thiobarbituric acid reaction substances (TBARS) assay were used to determine whether HAs could suppress oxidation of LDL stimulated by Cu^2+^, and ox-LDL-induced RAW 264.7 cells were used to research the protective of HS on RAW 264.7 cells. The results showed that HS (1, 1.5, 2 mg/mL) effectively inhibited the oxidation of LDL, and reduced apoptosis of RAW 264.7 cells. Besides, two components were identified by HPLC, namely, cyanidin and delphinidin.

*Hibiscus sabdariffa* L*.* leaves, the edible part of *H. sabdariffa* L., consist of multiple polyphenols, and possess a variety of pharmacological functions, including anti-hyperglycaemia, anti-hyperlipidemia, and anti-oxidation. In 2013, Chen et al. (Chen et al., 2013) studied whether *H. sabdariffa* L. leaf polyphenolic extract (HLP) could inhibit the formation of foam cells. The ability to inhibit LDL oxidation of HLP (mainly including catechin, (−)-epicatechin gallate, ellagic acid, ferulic acid, and quercetin) was measured by TBARS analysis, agarose gel electrophoresis and electrophoresis of Apo B fragmentation assay. The results showed that HLP could inhibit LDL oxidation. Subsequently, the Oil Red O staining assay was utilized to study the effect of HLP on foam cell formation, and the result indicated the amount of foam cells was increased with treatments of HLP. In addition, the results of WB indicated that the underlying mechanism was up-regulating LXRa/ABCA1 pathway and decreasing the expressions of CD36 and PPAR-γ.

#### 5.2.2 *Scoparia dulcis* L

Due to the ability of anti-diabetes, anti-hypertension, anti-hyperlipidemia, and anti-tumor, *Scoparia dulcis* L., an edible ethnomedicinal folklore botanical drug, has attracted the interest of researchers. It has been reported that *S. dulcis* L. could reduce levels of LDL cholesterol in diabetic rats with its anti-oxidative and anti-inflammatory effects, so that *S. dulcis* L. might have an anti-atherosclerotic effect (Pari and Latha, 2006). In 2014, Nambiar et al. (Nambiar et al., 2014) found that foliar methanol extract of *S. dulcis* effectively inhibited lipid peroxidation and LDL oxidation, indicating that *S. dulcis* L. might own strongly anti-oxidative effect. In addition, *S. dulcis* L. inhibited the formation of ox-LDL, as shown in the result of the Oil red O staining. Moreover, *S. dulcis* L. improved human erythrocyte membrane stability, suggesting anti-inflammatory effects of *S. dulcis* L. Besides, myricetin and rutin, identified by HPLC, might be the main components of *S. dulcis* L.

#### 5.2.3 *Phyllanthus emblica* L

*Phyllanthus emblica* L., belonging to the Phyllanthaceae family, is an edible fruit distributed in many countries, and is also used as a medicinal fruit to prevent diseases. Traditionally, *P. emblica* L. is believed to have antioxidant and anti-aging effects. And then, the anti-inflammatory, hypoglycemic, hypolipidemic, and immunomodulatory effects of *P. emblica* L. were proved by modern research. It has been reported that *P. emblica* L. could decrease cholesterol levels in cholesterol-fed rats, showing anti-atherosclerotic effects (Kim et al., 2005). Then, Nambiar et al. (Nambiar and Shetty, 2015) studied the effects of *P. emblica* L. on LDL oxidation and foam cell formation, and found that *P. emblica* L. on the concentration of 50 μg/mL effectively inhibited the oxidation of LDL induced by Cu^2+^, and reduced uptake of ox-LDL in RAW264.7 cells to inhibit foam cell formation. In addition, the results of HPLC analysis showed that phenolic and flavonoid are the main component groups of *P. emblica* L., while kaempferol, rutin, and coumaric acid are the three components with the highest content.

### 5.3 Whole extract (aqueous and ethanol extract)

#### 5.3.1 *Morus alba* L

Mulberry leaf, the leaf of *M. alba* L., is a traditional Chinese medicine that has been used for anti-diabetes, anti-hyperlipidemia, and prevention of coronary artery disease (CAD) for many years (Andallu et al., 2001). Yang et al. (Yang et al., 2011). Studied the effect of mulberry leaf polyphenolic extracts (MLPE) on LDL oxidation and foam cell formation, and found that MLPE could attenuate the oxidation and lipid peroxidation of LDL, reduce ox-LDL-generated ROS, and elevate the pressions of SOD-1 and GPx in macrophages, suggesting that MLPE had a strongly anti-oxidative effect. Furthermore, MLPE effectively inhibited the foam cell formation and decreased the amount of TG and cholesterol, and the underlying mechanism might be downregulating expression of expression of PPARγ, CD36, and SR-A, implying that MLPE reduced the uptake of ox-LDL. According to the results of HPLC analysis, gallocatechin, gallocatechin gallate, and naringenin are the main components.

#### 5.3.2 *Trachyspermum ammi* (L.) sprague

*Trachyspermum ammi* (L.) Sprague (*Trachyspermum ammi*) is a traditional Chinese medicine and a very famous spice, which is usually used in respiratory ailments, bronchial pneumonia, and stomach disorders. In recent years, it has been reported that *T. ammi* has antioxidant, antihyperlipidemic, and antidiabetic effects. In addition, honey, a natural sweetener used in many countries, is beneficial in preventing cardiovascular diseases, including AS. However, the anti-atherosclerotic effects of the combination of *T. ammi* and honey had not been demonstrated. Therefore, Priyaa et al. studied how the extracts of *T. ammi* and honey work on AS, especially on antioxidation and lipid metabolism (Priyaa and More, 2022). In their results, tannin methanol extract (TME) showed the highest activity to inhibit LDL oxidation and further inhibited ox-LDL-induced foam cell formation, apoptosis, and proliferation. Moreover, the analysis of TEM compositions *via* HPLC and GC-MS showed 2-(4-Methyl-1H-1,2,3-triazol-1-yl) ethan-1-amine, 1,3-Dioxolan-2-one, 4,5-bis (methylene)-, N-methylene-n-octadecylamine are the three ingredients with the highest content.

### 5.4 Others

#### 5.4.1 *Pinus morrisonicola* hayata

*Pinus morrisonicola* Hayata, a plant of the Pinaceae family, has a variety of pharmacological effects, including anti-oxidation, anti-mutagenicity, and anti-inflammation, and has been used as a folk medicine for anti-hypertension in Asia for a long time. In 2008, Yen et al. (Yen et al., 2008) reported that *P. morrisonicola* Hayata could inhibit Cu^2+^-mediated LDL oxidation and increase NO production in cells, showing an anti-atherosclerotic effect. Considering the important role of ox-LDL-induced foam cells in AS development, in 2015, Cheng et al. (Cheng et al., 2015) studied the effects of essential oil extracted from *P. morrisonicola* Hayata (PME) on ox-LDL-induced RAW264.7 cells. The results showed that PME contained 1-docosene, neophytadiene, and methyl abietate, and could inhibit lipid peroxidation and foam cell formation *in vitro*. Therefore, it seems that PME has an anti-atherosclerotic effect through its anti-oxidative capacity.

#### 5.4.2 *Citrus × aurantium f. deliciosa* (ten.) M.Hiroe

Recently, accumulating evidence has suggested that medicinal essential oils usually own properties of anti-oxidation and lowering blood lipid, but the molecular mechanism still has been unclear. Mandarin [*Citrus × aurantium f. deliciosa* (Ten.) M. Hiroe] peel oil (MPO) is recognized to be safe and has an anti-atherosclerotic effect (Chung et al., 2007; Chung et al., 2008). In 2020, Castro et al. (Castro et al., 2020) studied the effects of MPO on cholesterol metabolism and lipid synthesis, and its antioxidant capacity. The results showed that MPO (mainly includes limonene) decreased cholesterol synthesis *via* inhibiting post-squalene reaction of the mevalonate pathway, while inhibited LDL oxidation under oxidative stress. In addition, MPO inhibited foam cell formation and significantly decreased lipid quantity in foam cells, and downregulated the expression of CD36. In a word, the above results suggest that MPO may be a potential candidate for preventing AS.

### 5.5 Monomers

#### 5.5.1 Curcumin

Curcumin is a bioactive compound derived from the rhizomes of *Curcuma longa* L., and has been reported to possess a variety of activities, such as anti-tumor and anti-inflammatory. In 2013, Kou et al. (Kou et al., 2013) studied the effect of curcumin on Cu^2+^-mediated LDL oxidation in a cell-free system and foam cell formation, and found that curcumin (10 μM) could increase the level of MDA and dose-dependently attenuate LDL oxidation. Later, Min et al. (Min et al., 2013) studied the potential molecular mechanism of curcumin to inhibit foam cell formation, and their results showed that the expression of CD36 and PPAR-γ was downregulated, and the phosphorylation of p38 MAPK was inhibited in ox-LDL-induced RAW 264.7 cells, suggesting that curcumin reduced the formation of ox-LDL *via* inhibiting the phosphorylation of p38 MAPK.

#### 5.5.2 (3R)-5-hydroxymellein

(3R)-5-Hydroxymellein is a secondary metabolite from a plant called *Vitex rotundifolia* L. f. From the first time (3R)-5-Hydroxymellein was isolated and identified in 1990 until now, it has been reported to have a variety of pharmacological activities, such as antifungal, antibacterial, and antioxidant effects (Wedge and Nagle, 2000). In 2020, Kim et al. (Kim et al., 2020) first illustrated that (3R)-5-Hydroxymellein might have the ability to reduce the risk of AS. In their research, the production of conjugated dienes and malondialdehyde, the amount of hyperchromicity and carbonyl content, and anti-LDL oxidation were used to demonstrate the oxidation of LDL and HDL, while the Oil red O staining was used to demonstrate the inhibition of foam cell formation. The results suggested that (3R)-5-Hydroxymellein could inhibit the oxidation of LDL and HDL, as well as inhibit foam cell formation.

#### 5.5.3 Maslinic acid

Maslinic acid (MA) is a natural pentacyclic triterpene and can be isolated from many natural sources, including herbal remedies, vegetables, and fruits. MA has various beneficial properties, such as antioxidant, anti-inflammatory, antitumoral, and cardioprotective effects (Liu et al., 2011). Thus, Phang et al. (Phang et al., 2020) hypothesized that MA could inhibit foam cell formation, and verified it by a series of experiments *in vitro*. In detail, the Oil Red O staining and flow cytometric analysis were utilized to explore the formation of foam cells, while Cu^2+^-stimulated LDL was utilized to explore LDL oxidation, and the results showed that MA (20 μM) inhibited LDL oxidation and foam cell formation. In addition, MA reduced THP-1monocyte adhesion to HUVEC cells *via* downregulated the expression of VCAM-1 and MCP-1, and enhanced cholesterol efflux *via* downregulated the expression of SR-A and CD36, while upregulated the expression of ABCA1 and ABCG1. In conclusion, MA showed a strong ability for AS prevention.

#### 5.5.4 Lusianthridin

Lusianthridin (LST) is a phenanthrene compound isolated from *Dendrobium venustum* Teijsm. & Binn., which belongs to the family Orchidaceae. As reported in a research article, LST was a potential antioxidant. In thalassemia patients, an oxidant called hemin can be found, which causes oxidative stress and LDL oxidation, and is a risk factor for AS. In 2021, Thant et al. (Thant et al., 2021) verified whether LST could attenuate hemin-induced ox-LDL and foam cell formation. In their study, results of TBAR assay and REM assay suggested that LST inhibited LDL oxidation, while the result of the Oil Red O staining assay suggested that LST inhibited ox-LDL-induced foam cell formation. Otherwise, LST improved the level of cholesteryl arachidonate and cholesteryl linoleate.

## 6 Conclusion and perspectives

AS is a chronic disease with complex pathogenesis, and lipid metabolism disorder is one of the important mechanisms of AS pathogenesis, which makes great contributions to occurrence and development of AS. It is found that metabolic disorder of cholesterol, an important lipid component in the human body, is closely related to oxidative stress (Khatana et al., 2020). As mentioned above, fat-soluble cholesterol cannot be transported in the blood alone. Therefore, it must combine with apolipoprotein and other components to form plasma lipoprotein. LDL, composed of cholesterol ester, triglyceride, phospholipid, and apolipoprotein, is the plasma lipoprotein with the highest cholesterol content and plays an important role in cholesterol metabolism (Itabe, 1998). Under normal physiological conditions, cholesterol is transported to peripheral tissues by LDL, and then is reversely transported to the liver by HDL. Finally, part of the cholesterol is reabsorbed, while the other part is discharged from the body. However, under the condition of oxidative stress, LDL containing a large number of cholesterol esters is oxidized to ox-LDL, which causes macrophages to engulf a large number of ox-LDL and eventually form foam cells, promoting the development of AS (Braamskamp et al., 2015). In addition, ox-LDL can promote the development of AS in many ways, including causing endothelial dysfunction, promoting smooth muscle cell migration and proliferation, and activating platelets. Therefore, inhibiting the oxidative modification of LDL is of great significance for the prevention and treatment of AS.

It has been a long time since herbal medicines to be used to prevent and treat diseases, and herbal medicines have made great contributions to human health. And there are accumulating studies have reported that botanical drugs and their extracts have significant effects on AS, including protecting vascular endothelial cells, improving lipid metabolism disorder, reducing foam cell formation, and so on (Zhang et al., 2021). In recent years, more and more studies have reported that botanical drugs and their extracts can significantly inhibit the oxidation of LDL, thereby reducing the production of foam cells to play an anti-atherosclerosis role. In these researches, copper ions have been used to oxidize the separated LDL *in vitro*, while REM, fragmentation of Apo B, and TBARS assay are used to measure whether LDL is oxidized. In addition, RAW 264.7 cells induced by ox-LDL have been used for Oil red O staining to judge the generation of foam cells. Moreover, PCR and WB experiments have been used to reveal the potential molecular mechanism of herbal medicines to treat AS. The results showed that herbal medicines mentioned in this review had significant antioxidative effects and could effectively inhibit the oxidation of LDL. In addition, these herbal medicines regulated cholesterol homeostasis by reducing cholesterol intake and increasing its efflux, and the molecular mechanism was related to down-regulating the expression of scavenger receptor CD36 and SR-A, and up-regulating the expression of ABCA1 and ABCG1. Interestingly, most of the effective ingredients reported in the articles collected in this review are phenols and polyphenols extract of herbal medicines. Phenols/polyphenols are compounds with one aromatic ring attached to one or more hydroxyl functional groups in structure, and widely exist in fruits, vegetables, botanical drugs, and tea. It has been reported that phenols/polyphenols have strong antioxidant activity, which may improve cardiovascular diseases and neurodegenerative diseases (Cheng et al., 2017). This review summarized the antioxidation of LDL and inhibition of foam cell formation effects of phenols/polyphenols extracts from mulberry leaf, *H. sabdariffa* leaf, *Eugenia jambolana* seed, mulberry anthocyanin, *S. dulcis*, *Emplica officinalis*, lusianthridin, and *T. ammi*.

Although a large number of studies have clarified to some extent that herbal medicines can prevent and cure AS by inhibiting LDL oxidation, some deficiencies still exist. First, most of current studies measured *in vitro* experiments, therefore, the beneficial effects and safety *in vivo* should be clarified. Especially, the change of LDL and ox-LDL levels after treatment with herbal medicines should be determined *in vivo*. Second, existing research has not revealed the molecular mechanisms of these herbal medicines, thus, in-depth research should be carried out to find underlying molecular mechanisms. Third, current research mostly used mixed extracts of herbal medicines, and individual compounds that play pharmacological roles were not clear, limiting the further development of these herbal medicines. In addition, some active compounds are found in very low levels in plants, while sources of these active compounds have not been systematically developed. So, it is necessary to find more sources. Fourth, toxicity studies on these herbal medicines are still lacking, so a lot of subsequent work is needed to determine target organs, toxic reactions and mechanisms, and to explore detoxification methods to ensure the safety of these herbal medicines. Fifth, due to complex components and unexplained mechanisms, the promotion of herbal medicines is limited. Although herbal medicines have gained widespread clinical applications in some Asian countries, they are rarely used in many western countries worldwide. Therefore, more clinical trials need to be measured to verify the safety and effectiveness of herbal medicines. In future research, these problems need to be solved. Sixth, current research on these herbal medicines is focused on pharmacodynamic studies, and appropriate drug delivery system should be developed.

In conclusion, this review described the effects of lipid metabolism disorder related to LDL oxidation on AS and the beneficial role of herbal medicines in this process. We hope that this review will provide ideas for the development of medicines for the prevention and treatment of AS from herbal medicines.

## Glossary

ABCA1: ATP-binding cassette transporter A1
ABCG1: ATP-binding cassette protein G1
ACAT1: acetyl-CoA acetyltransferase
AGEs: advanced glycation end products
AS: atherosclerosis
ATH: atherogenic diet
CAD: coronary artery disease
CD36: scavenger receptor B2
Chol-HPODE: cholesteryl hydroperoxy-octadecadienoate
CM: chylomicrons
EDRF: endothelium-dependent hyperpolarization factor
EGF: epidermal growth factor
ER: endoplasmic reticulum
ET-1: endothelin-1
HDL: high-density lipoprotein
HLP: *H. sabdariffa* leaf polyphenolic extract
HS: *Hibiscus sabdariffa* L.
IDL: intermediate-density lipoprotein
IGF-1: insulin-like growth factor
LAL: lysosomal acid lipase
LDL: low density lipoprotein
LOX-1: lectin-like ox-LDL receptor-1
LST: lusianthridin
LXR: liver X receptor
MA: maslinic acid
MACs: mulberry anthocyanin-rich extracts
MLPE: mulberry leaf polyphenolic extracts
mmLDL: minimally modified LDL
MMP: matrix metalloproteinase
MPO: mandarin (*Citrus reticulata*) peel oil
NADPH: nicotinamide adenine dinucleotide phosphate
NCHH: neutral cholesterol ester hydrolase
NPC1L1: Niemann-Pick type C1-like 1
NO: nitric oxide
PBLs: piper betel leaves
PDGF: platelet derived growth factor
PGI2: prostacyclin
RCT: reverse cholesterol transport
REM: relative electrophoretic mobility
ROS: reactive oxygen species
SR-A: scavenger receptor class A
SR-B1: scavenger receptor class B type 1
SSE: *Syzygium cumini* (L.) Skeels seed extract
TBARS: thiobarbituric acid reaction substances
TME: tannin methanol extract
VLDL: very low-density lipoprotein
VSMCs: vascular smooth muscle cells
